# Clinical Theragnostic Relationship between Chemotherapeutic Resistance, and Sensitivity and miRNA Expressions in Head and Neck Cancers: A Systematic Review and Meta-Analysis Protocol

**DOI:** 10.3390/genes12122029

**Published:** 2021-12-20

**Authors:** Peter Shaw, Greg Raymond, Raghul Senthilnathan, Chellan Kumarasamy, Siddhartha Baxi, Deepa Suresh, Sameep Shetty, Ravishankar Ram M, Harish C. Chandramoorthy, Palanisamy Sivanandy, Suja Samiappan, Mogana Rajagopal, Sunil Krishnan, Rama Jayaraj

**Affiliations:** 1 Department of Artificial Intelligence, Nanjing University of Information Science and Technology (NUIST), Nanjing 210044, China; 100001@nuist.edu.cn; 2 Menzies School of Health Research, Darwin, NT 0810, Australia; 3 Northern Territory Medical Program, CDU Campus, Flinders University, Ellengowan Drive, Darwin, NT 0909, Australia; greg.raymond@flinders.edu.au; 4 School of Biosciences and Technology, Vellore Institute of Technology (VIT), Vellore 632014, India; Raghulsnn@gmail.com; 5 Kumarasamy School of Health and Medical Sciences, Curtin University, Perth, WA 6102, Australia; chellank54@gmail.com; 6 MBBS, FRANZCR GAICD (SB), Genesis Care Gold Coast Radiation Oncologist, Southport, QLD 4224, Australia; Siddhartha.baxi@genesiscare.com; 7 Division of Endocrinology, Department of Internal Medicine, Mayo Clinic Florida, Jacksonville, FL 32224, USA; Deepa.Suresh@mayo.edu; 8 Department of Oral and Maxillofacial Surgery, Manipal College of Dental Sciences, Mangalore, Academy of Higher Education, A Constituent of MAHE, Manipal 576104, India; sameep.shetty@manipal.edu; 9 Department of Pharmaceutical Biology, Faculty of Pharmaceutical Sciences, UCSI University Kuala Lumpur (South Wing), No.1, Jalan Menara Gading, UCSI Heights 56000 Cheras, Kuala Lumpur 57000, Malaysia; ravishankar@ucsiuniversity.edu.my (R.R.M.); mogana@ucsiuniversity.edu.my (M.R.); 10 Department of Microbiology and Clinical Parasitology, College of Medicine, King Khalid University, Abha 61421, Saudi Arabia; hshkonda@kku.edu.sa; 11 Department of Pharmacy Practice, School of Pharmacy, International Medical University, Bukit Jalil, Kuala Lumpur 57000, Malaysia; PalanisamySivanandy@imu.edu.my; 12 School of Postgraduate Studies, International Medical University, Bukit Jalil, Kuala Lumpur 57000, Malaysia; 13 Department of Biochemistry, Bharathiar University, Coimbatore 641046, India; sujaramalingam08@gmail.com; 14 Department of Radiation Oncology, Mayo Clinic Florida, 4500 San Pablo Road S., Jacksonville, FL 32224, USA; krishnan.sunil@mayo.edu; 15 Northern Territory Institute of Research and Training, Darwin, NT 0909, Australia

**Keywords:** head and neck cancer, miRNA, chemoresistance, protocol, systematic review, hazard ratio, patient survival, up-regulation, down-regulation

## Abstract

**Background:** The microRNAs (miRNAs) are small noncoding single-stranded RNAs typically 19–25 nucleotides long and regulated by cellular and epigenetic factors. These miRNAs plays important part in several pathways necessary for cancer development, an altered miRNA expression can be oncogenic or tumor-suppressive. Recent experimental results on miRNA have illuminated a different perspective of the molecular pathogenesis of head and neck cancers. Regulation of miRNA can have a detrimental effect on the efficacy of chemotherapeutic drugs in both neoadjuvant and adjuvant settings. This miRNA-induced chemoresistance can influence the prognosis and survival rate. The focus of the study is on how regulations of various miRNA levels contribute to chemoresistance in head and neck cancer (HNC). Recent findings suggest that up or down-regulation of miRNAs may lead to resistance towards various chemotherapeutic drugs, which may influence the prognosis. **Methods:** Studies on miRNA-specific chemoresistance in HNC were collected through literary (bibliographic) databases, including SCOPUS, PubMed, Nature, Elsevier, etc., and were systematically reviewed following PRISMA-P guidelines (Preferred Reporting Items for Systematic Review and Meta-analysis Protocol). We evaluated various miRNAs, their up and downregulation, the effect of altered regulation on the patient’s prognosis, resistant cell lines, etc. The data evaluated will be represented in the form of a review and meta-analysis. **Discussion:** This meta-analysis aims to explore the miRNA-induced chemoresistance in HNC and thus to aid further researches on this topic. PROSPERO registration: **CRD42018104657**.

## 1. Introduction

Head and Neck Cancers is a collective term for cancers that usually originate in the squamous cell lining of the mucosal membrane of the inner mouth, nose, and throat [1]. Micro RNAs are a class of small, endogenous, noncoding, 18–24 nucleotide long sequences that play a crucial role in many processes essential for cancer progression, including cell death, proliferation, metastasis, and treatment resistance [2,3,4,5,6]. Recent studies show that chemoresistance to specific chemotherapeutic drugs such as Cisplatin, Doxorubicin, 5-fluorouracil, etc., is caused due to varying regulation of specific miRNAs [7]. These miRNAs can reduce the efficacy of drugs or even make cancer cells utterly impervious to the drug [8,9,10,11,12,13,14,15,16,17,18,19,20,21,22,23,24]. Such resistance can significantly worsen the prognosis of HNC patients and can also reduce survival rates. Adjuvant therapy is rendered inefficient, and patients show poor responses towards it. This study evaluates the chemoresistance due to miRNA at various stages of HNC.

HNC ranks as the sixth most prevalent type of cancer in the world. It is a heterogeneous disease with various categories based on anatomical location, etiology, and molecular characteristics [25,26]. Head and neck cancer accounts for more than 931,000 cases and 467,000 deaths annually worldwide [27]. Studies have shown that males are affected significantly more than females, with a ratio ranging from 2:1 to 4:1 [28,29]. Head and neck squamous cell carcinomas (HNSCCs) occur in the oral cavity, pharynx, or larynx and account for the bulk of HNCs [26]. Excessive use of tobacco and alcohol, as well as infection with the human papillomavirus (for oropharyngeal cancer) and Epstein-Barr virus, are all etiological factors in the development of HNSCCs (for nasopharyngeal cancer) [26]. Malignant proliferation and chemoresistance continue to be the limiting factors in HNC treatment, which leads to loco-regional relapse or distant metastasis [30,31]. Current therapy options for HNC include surgery, radiotherapy, chemotherapy, and most recently, anti-EGFR antibody treatment [32].

The focus of this study protocol is to describe the quantitative and qualitative effects of miRNA-specific chemoresistance in head and neck cancer. The primary objective of this systematic review is to explore the influence of miRNA expressions in head and neck carcinoma patients by assimilating and evaluating the evidence. The secondary objective of this proposed study is to assess the deranged regulation of miRNAs and evaluate their resistance in cell lines which may cause recurrence.

## 2. Rationale

Most of the publications on miRNA-specific chemoresistance of HNC are particular to evaluating the effects of specific miRNA. Published studies have been specific to the samples collected in a hospital related to a particular geographical region and were localized to that area. This systematic review and meta-analysis aims to integrate the data obtained by the publications as mentioned earlier and present it in a peer-reviewed and extensively evaluated fashion. This systematic review has been proposed after a thorough evaluation of studies and publications from various countries and hence is not restricted to the studies performed in a specific region.

This study will evaluate the data collected on various miRNAs, focusing on how their regulation can affect the sensitivity of a chemotherapeutic drug. The reviewers hope the meta-analysis will help researchers get a better perspective on how miRNA is linked to chemoresistance towards various chemotherapeutic drugs. The comprehensive meta-analysis provides quantitative synthesis of currently existing studies on miRNA specific chemoresistance in HNA patients.

This study collates risk factors from various studies and provides a broad overview of miRNA-induced chemoresistance.

### Review Questions

This systematic review protocol aims to explain the methodological approaches implemented in conducting this meta-analysis on miRNA Specific Chemoresistance in HNC. The study proposes the following questions:

1. What effect does miRNA regulation have on chemotherapy?
2. What is the general prognosis of patients having miRNA-specific chemoresistance?
3. What are the miRNAs most responsible for chemoresistance in HNC patients?
4. What are the survival rates associated with each miRNA linked to chemoresistance, and how are they affected?
5. How does the prognosis vary for different miRNA causing the chemoresistance?
6. What is the future of miRNA as an active form of cancer treatment for Head and Neck Cancer?

## 3. Materials and Methods

This study aims to evaluate the effect of miRNA expression in HNC patients, reporting this particular kind of resistance worldwide on prognosis. The research protocol will be conducted in accordance with the Preferred Reporting Items for Systematic Reviews and Meta-Analyses (PRISMA) guidelines [33]. This study aims to detect and analyze the miRNA-specific chemoresistance in head and neck carcinoma. This study is neither confined to any datasets nor a particular region.

### 3.1. Search Methods

The study clearly defines the effect of miRNA expression in HNC patients and the altered up or down-regulation of miRNA in the prognosis of patients. This systematic review will also include those studies examining miRNA specific chemoresistance in HNC patients. This study will utilize a comprehensive search strategy based on the keywords listed in Table 1. The study will begin with an initial limited search of online bibliographic databases such as EMBASE, PubMed, Elsevier, Science Direct, SCOPUS, Nature, and Web of Science. This search will be then be expanded to include the words contained in the title and abstract in the papers considered in the analysis. There will be no restrictions on study participants in terms of age, gender, ethnicity, country of origin, and morbidities (for patients and the general population). The search will be limited to articles published between 2000 and 2021 to focus mainly on recent advancements in research outcomes in this field. The next level of the search will be a detailed manual search of the full-text articles to gather and retrieve all the required information for the systematic review from the bibliographic articles. 

The reviewers will discard any full-text studies that do not meet the inclusion criteria. Finally, a manual search of selected articles will be conducted to extract more research from references. If there are any differences among the reviewers, they will be resolved through discussion or with the help of an unbiased third reviewer.

### 3.2. Selection Criteria

#### 3.2.1. Inclusion Criteria

- Studies analyzing the effect of miRNA expressions in both HNC patients and cell lines will be considered.
- Studies analyzing miRNAs and resistance/are performed in liquid biopsies (plasma, saliva....) will be included.
- Studies that discuss the clinicopathological characteristics of HNC patients along with hazard ratio or Kaplan–Meier curve will be included.
- Studies reporting resistance in HNC will be included.
- Articles that discuss the survival outcomes of almost all stages of HNC patients will be included in the meta-analysis.
- Studies reporting miRNA profiling platform and miRNA expressions analysis using in vitro assays will be included.
- Studies that differentiate between 3p and 5p in the microRNAs expressions in HNC
- Genes and/or pathways involved in chemoresistance or sensitivity in HNC patients will also be considered.
- miRNA expression analysis, HR, and associated 95% CI or Kaplan–Meier (KM) curve is required for the eligible studies.
- Studies appropriate to PRISMA guidelines for systematic review and meta-analysis will be included.

#### 3.2.2. Exclusion Criteria

- Studies published in languages other than English.
- Any information or results from letters to the editors, case studies, conference abstracts, case reports, and review articles of HNC will be removed.
- Studies performed only in vitro will be excluded and will not be considered for the systematic review.
- Studies in which proper discussion about miRNA profiling and pathways related to that are not available will be excluded.
- Studies with no accessibility to survival outcomes, HR values, or Kaplan–Meier (KM) curve will not be considered for the meta-analysis.
- Studies using patients’ information from datasets or cancer registries will be removed.
- Studies whose full texts are not accessible will also be excluded.
- Duplicates will be removed, and the study will be excluded if it falls within the exclusion criteria.

#### 3.2.3. Participants

The systematic review and meta-analysis will add studies involving patients suffering from all types of HNC. Participants with evidently established diagnoses of HNC will be included. There will be no restrictions on study participants in terms of age, gender, ethnicity, country of origin, and morbidities (for patients and the general population). 

### 3.3. Data Collection and Management

The data collected by the reviewers will be saved in a Microsoft Excel spreadsheet. The data will be integrated with pertinent material, with repeats removed and reviewed later. Only full-text articles that meet the inclusion and exclusion criteria will be retrieved. The following data will be collected from the selected papers:

- Author name and information
- Type of head or neck cancer
- Date and journal of publication
- Type of micro-RNA studied
- Hazard Ratio and 95% Confidence Interval
- Patients origin (by country)
- Type/origin of Samples collected for analysis
- The total number of samples used
- Cell lines and the pathways affected
- Clinical stage of the affected HNC patients and their details
- Type of chemoresistant cells and chemoresistant drug
- Micro-RNA profiling platform

#### Data Items Included in this Study

- Characteristics of study material (author names, geographical area of study, type of study, year of publication)
- Characteristics of study participants (country of origin, clinical stage of cancer, type of expressed miRNA, drug to which resistance is expressed)
- Characteristics of study methods and results (miRNA profiling platform, number of samples, statistical analysis)

### 3.4. Study Selection Process

The reviewers will initially analyze abstracts and titles retrieved through the primary search approach against the selection criteria individually. Full re-reports will be obtained for any titles that appear to fulfill the study selection criteria. Second, the reviewers will examine the full-text articles to acquire research-related information and address any issues about the eligibility of the selected articles. Any study that fails to fulfill the required selection criteria will be excluded. The writers will additionally look for extra material in the references of the chosen publications to ensure that all of the necessary information for the study is gathered and nothing vital is overlooked. 

**Figure 1:**
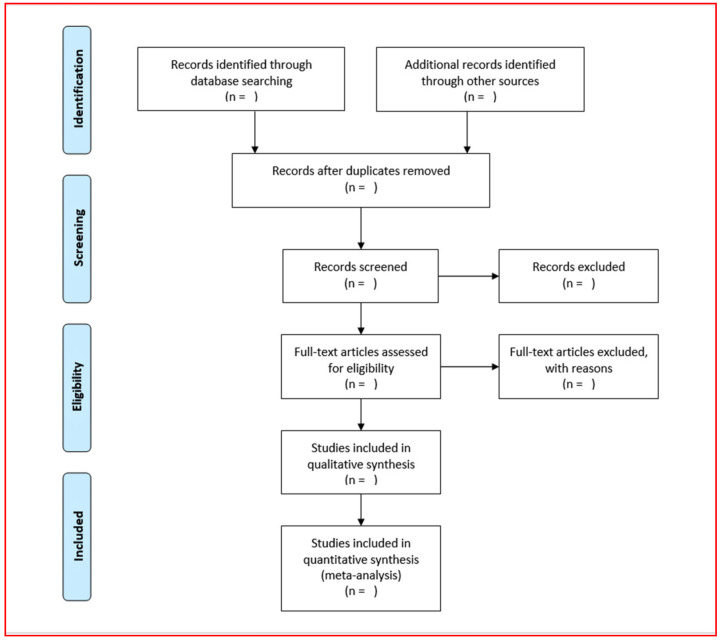
Schematic representation of the selection process.

Any divisive viewpoint will be settled through discussion. The reviewers will record the reasons for excluding studies. Neither of the review authors will be blind to the journal titles or the study authors and their respective institutions. The quality appraisal factors listed below will be assessed, graded, and documented: the author’s name, sample size, patient age, and gender, country of origin, disease stage, miRNA expression, study period, survival outcome, miRNA profiling platform, gene/pathways related, statistical data, drugs or chemotherapy, resistant cell lines, outcome variables, and other factors. The search results will be published following PRISMA criteria. A PRISMA flow chart will be utilized to summarize the selection procedure used to filter through the studies initially gathered (Figure 1).

### 3.5. Study Outcomes

Primary outcome: The study’s primary outcome is to evaluate the miRNA expression in the head and neck cancer patients, gender standardized prevalence of the disease, and the survival outcome rate linked with HNC patients.

Secondary outcome: The study’s secondary outcome is to analyze the effect of miRNA-specific chemoresistance in HNC patients. It also aims to investigate the survival rates associated with each miRNA linked to chemoresistance and how they are affected.

### 3.6. Mitigating Risk of Bias in Individual Studies

The National Heart, Lung, and Blood Institute’s (NHLBI) quality evaluation tool for observational and cross-sectional studies will be utilized to assess the quality of selected studies [34]. This tool will be used to rate all full-text articles classified as good, fair, or bad. The risk of bias in the studies will be assessed by reviewers. Each reviewer will work independently and should have the needed expertise to determine the validity of the studies collected based on factors such as the number of samples collected, the number of patients, the miRNA profiling platforms used, the year of publication, the duration of the study, and geographical area analysis. Other considerations will be evaluated if necessary.

### 3.7. Data Synthesis

Each author will gather information from several databases. The data collected will be collated and sent to the reviewers following data collection. Then, data will be extracted and tabulated to determine the type of survival (OS, DFS, DSS, PS, and LCR), type of miRNA, HR, and CI. The collected data will be utilized to create statistical results for all of the studies included in the meta-analysis, such as the pooled HR and CI. The statistical data for the study will be generated using the ‘Comprehensive Meta-Analysis Soft-ware.’ The miRNA will be analyzed and classified based on expression (up-regulation and down-regulation).

### 3.8. Meta-Analysis and Subgroup Analysis

Meta-analyses for this particular study will be conducted using “Comprehensive Meta-Analysis V.3.0” software for the HR and 95% CI values obtained from the relevant materials collected for this study. The pooled HR value as an estimated effect size provides more clinical utility as it examines the survival pattern of the patients of the included studies. Heterogeneity will be calculated using Tau Square, Cochran’s Q test [35] and I^2^ statistic [36]. These parameters increased robustness in analysis of between study heterogenicity. Additionally, Z statistics will be used to calculate the heterogeneity. 

The I^2^ statistic will be used to assess the degree of heterogeneity between studies, with an I^2^ value more than 50% indicating considerable heterogeneity. The meta-analysis will use a random or fixed effects model based on the heterogeneity. A P value of less than 0.01 will be considered statistically significant for the Q test. In the meta-analysis, the z-test will also be used to estimate how many standard deviations each research can deviate from the study mean. To detect heterogeneity, the Eggers bias indicator test will be performed [37]. Quality assessment and statistical analysis would be performed [34]. CMA will be used to compute the pooled HR and 95% CI.

Publications bias will be evaluated using Orwin and classic fail-safe N test (demonstrates the likelihood that studies are absent from the current meta-analysis and these studies if included in the analysis, would shift the Hazard Ratio of the included studies toward the null) [38], Egger’s bias indicator test (gold standard regression test), Begg and Mazumdar Rank collection test (defines the estimated or computed Tau between Hazard Ratio and standard error), Duval and Tweedie’s trim and fill calculation (explores the missing studies that likely to fall, adds them to the analysis and then recommutes the pooled HR) [39], and inverted funnel plot. The inclusion of these publication bias indicators will explain the possible publication bias from small or missing studies.

Subgroup analysis will be performed considering location or origin of the reported incidence, anatomical sites of cancer growth, variation and resistance to HNC among different age groups and different genders. Subgroup analysis will be performed on all miRNAs found to be differentially expressed in the studies. Different miRNA expression patterns in HNC will be investigated, as will their influence on the development of chemoresistance. Subgroup analysis will be performed based on the studies that differentiate between 3p and 5p in the microRNAs expressions in HNC.

### 3.9. Reporting of the Review

The PRISMA 2015 guidelines will be followed when reporting and communicating the results of this study. A flow diagram indicating the selection process of selected studies will be made. These results also include detailed inclusion and exclusion criteria to aid in the selection process of the appropriate material. A search strategy will also be included to identify the required data. The collected data such as HR and 95% CI values will be used to create Forest plots. Risk of bias, Publication bias, and Heterogeneity tests will be calculated to evaluate the validity and accuracy of the data collected.

## 4. Discussion

The pathophysiology underlying chemotherapy and polydrug resistance in humans involving polymorphic miRNAs play a vital role in regulating apoptosis, DNA patch-up, and epithelial-mesenchymal transition cell cycle regulation. 

This study focuses on evaluating the effect of miRNA on chemoresistance in HNC. It aims to determine the effect of the regulation of miRNA on chemoresistance. The collected HR and 95% CI will be used to create Forest plots. The outcome will be used to determine the effect of deregulation of miRNA on the prognosis of patients diagnosed with HNC. This systematic review and meta-analysis will provide a better understanding of the effects of miRNA on chemoresistance and prognosis.

Resistance to chemotherapeutic drugs is known to drastically deteriorate the prognosis of patients who show miRNA-specific chemoresistance. Many miRNAs can symbolize the growth of cancer by acting like biomarkers. For example, some miRNA from the ‘let 7-g’ family are thought to act as tumor suppressors, and an up-regulation of this miRNA can be used as a prognostic biomarker for detecting cancers [40].

The miRNA creates acquired chemoresistance by silencing genes/pathways that are directly or indirectly linked with the action of the chemotherapeutic drug. For example, in a study by Martz et al., the authors demonstrated that activation of the mitogen-activated protein kinase (RAS-MAPK), Notch-1, phosphoinositide 3-kinase (PI3K), and mammalian target of rapamycin (mTOR), PI3K/AKT, and estrogen receptor (ER) signaling pathways induced resistance in a selection of different drugs [41]. MiRNA dysregulation has an important role in modulating the principal mechanisms that induce HNC drug resistance that are currently known. Abnormal miRNA expression can disrupt the expression levels of multiple genes or important cellular pathways, which has a direct impact on the creation of chemotherapy resistance in HNC [42]. A study by Qin et al. clearly validates how the over expression of a miRNA interrupts the prevailing pathways and induce chemoresistance by directing the tumor suppressor genes [17]. As a result, it is critical to investigate the role of miRNA expression in controlling the prevalent pathways developing chemo resistance. It remains elusive to determine if the miRNA signature established for HNC can be replicated in future studies or for different tumor entities.

## 5. Ethics and Dissemination

This protocol was designed in accordance with the PRISMA-P guidelines. This study will be carried out using publically accessible data and will not include any human volunteers. As a result, no formal human research ethics committee assessment is required. Our findings will be published in peer-reviewed publications and conference proceedings. Furthermore, this study aims to provide a publicly reviewed standard for the systematic review with the expectation of maintaining standards. 

## Figures and Tables

**Table 1:** A sample keyword search strategy.

S No.	Search Items
1.	“miRNA” AND “treatment” OR drug resistance” AND “HNC” OR “Head and Neck Cancer”
2.	“microRNA” AND “drug resistance” AND “HNC” OR “Head and Neck Cancer”
3.	“Up-regulation OR down-regulation in HNC” OR “Differential Expression” OR “Deregulated miRNAs “OR “Head and Neck Cancer”
4.	“miRNA” AND “chemotherapeutic resistance” OR “chemosensitivity” AND “HNC” OR “Head and Neck Cancer”
5.	“miRNA” AND “treatment resistance” OR “chemoresistance” AND “HNC” OR “Head and Neck Cancer”
6.	“microRNA” AND “chemosensitivity” AND “HNC” OR “Head and Neck Cancer”
7.	“microRNA” AND “chemoresistance” AND “HNC” OR “Head and Neck Cancer”
8.	“HNC survival outcome” OR “Hazard Ratio” AND “HNC” OR “Head and Neck Cancer”

## Data Availability

Not applicable.
